# Caregiving in ALS – a mixed methods approach to the study of Burden

**DOI:** 10.1186/s12904-016-0153-0

**Published:** 2016-09-05

**Authors:** Miriam Galvin, Bernie Corr, Caoifa Madden, Iain Mays, Regina McQuillan, Virpi Timonen, Anthony Staines, Orla Hardiman

**Affiliations:** 1 School of Nursing and Human Sciences, Dublin City University, Dublin 9, Ireland; 2 Academic Unit of Neurology, Trinity Biomedical Sciences Institute, Trinity College Dublin, Dublin 2, Ireland; 3 Department of Neurology, National Neuroscience Centre, Beaumont Hospital, Dublin 9, Ireland; 4 Beaumont Hospital, Dublin 9, Ireland; 5 St Francis Hospice, Raheny, Dublin 5, Ireland; 6 School of Social Work and Social Policy, Trinity College Dublin, Dublin 2, Ireland

**Keywords:** Caregiver, Burden, Dimensions, Difficulties, Wellbeing, Amyotrophic lateral sclerosis, Motor neuron disease, Ireland, Mixed methods research

## Abstract

**Background:**

Caregiver burden affects the physical, psychological and emotional well-being of the caregiver. The purpose of this analysis was to describe an informal caregiver cohort (*n* = 81), their subjective assessment of burden and difficulties experienced as a result of providing care to people with Amyotrophic Lateral Sclerosis (ALS).

**Methods:**

Using mixed methods of data collection and analysis, we undertook a comprehensive assessment of burden and difficulties associated with informal caregiving in ALS. As part of a semi-structured interview a series of standardised measures were used to assess quality of life, psychological distress and subjective burden, and in an open-ended question caregivers were asked to identify difficult aspects of their caregiving experience.

**Results:**

The quantitative data show that psychological distress, hours of care provided and lower quality of life, were significant predictors of caregiver burden. From the qualitative data, the caregiving difficulties were thematised around managing the practicalities of the ALS condition, the emotional and psychosocial impact; limitation and restriction, and impact on relationships.

**Conclusions:**

The collection and analysis of quantitative and qualitative data better explores the complexity of caregiver burden in ALS. Understanding the components of burden and the difficulties experienced as a result of caring for someone with ALS allows for better supporting the caregiver, and assessing the impact of burden on the care recipient.

**Electronic supplementary material:**

The online version of this article (doi:10.1186/s12904-016-0153-0) contains supplementary material, which is available to authorized users.

## Background

Amyotrophic Lateral Sclerosis (ALS) also known as Motor Neuron Disease (MND) is a progressive, neurodegenerative disease, which impacts on the physical, communication and cognitive functioning of those affected. There is currently limited treatment and for the majority of patients death occurs within 3 years of symptom onset [1]. Management of ALS is palliative, treatment consists of symptom management and is aimed at maximising quality of life and minimising the burden of disease for patients and caregivers [2].

Care of people with ALS largely takes place in the community. Family caregivers are key figures in ALS care, and often play a central role in clinical decision making [3, 4]. Caring for a partner or family member with a progressive neurological illness has been recognised as being a source of burden and psychological distress, and impaired quality of life [5–9]. Time spent providing care and responsibilities of the caregiver generally increases with disease progression [10, 11]. Caregivers are confronted with short-term changes and long-term adjustments, increasing disabilities and levels of dependency, and the shifting nature of the care relationship [12].

Patient physical, cognitive and behavioural impairments can contribute substantially to the psychological and physical morbidity of the caregiver and affect caregiver burden in ALS [13, 14]. Effects on the caregiver’s health can be moderated by individual differences in resources and vulnerabilities, socioeconomic position, prior health status, and level of social support [15].

The term caregiver burden is frequently used but rarely deconstructed in ALS. The aim of this paper is to explore the multidimensional nature of caregiver burden in ALS through the use of quantitative and qualitative data, increasing the type and range of information available for analysis, for complementarity and additional coverage [16].

## Methods

### Research design

As part of a predominantly quantitative longitudinal study with smaller embedded qualitative components, the analysis reported in this paper uses a mixed methods approach to generate a broader variety of data that would not be accessible using quantitative or qualitative methods alone. Standardised measures and an open-ended question assessed aspects of caregiver burden in different ways.

### Participants

Caregiver participants were recruited as part of an ongoing longitudinal study of patients and their associated primary informal caregivers attending a specialist multidisciplinary ALS clinic in Beaumont Hospital, Dublin. While ninety patients had an associated caregiver at the time of the first interview, nine caregivers did not complete an open-ended question regarding difficulties associated with caregiving.

Accordingly, our analysis is based on the responses from 81 informal caregivers who provided both quantitative and qualitative data at their baseline interview, carried out between May 2013 and November 2014. This cohort was providing care to people who were both newly diagnosed, and at different stages in the disease trajectory, implying variation in duration of illness and associated implications for caregivers.

Written informed consent was obtained from all participants, some interviews were carried out during visits to the clinic but the majority took place in participants’ own homes, with the caregiver and interviewer present. Each interview lasted approximately 1 h. Confidentiality and anonymity were guaranteed.

### Assessment measures and data collection

During a semi-structured interview (Additional file 1), quantitative data were collected on a range of demographic and socio-economic variables. Caregiving in ALS has been shown to impact on caregiver psychological distress and quality of life [3, 7] and standardised validated measures commonly used in ALS caregiver research assessed Caregiver Burden (ZBI) [17], Psychological Distress (HADS) [18], and Quality of Life (MQol SIS) [19]. The assessment tools, measurement scales and cut-off scores are explained in Appendix 1, Table 1.

Qualitative data were collected from a single open-ended question during the interview to gain some insight into the respondent’s subjective definition of difficulties associated with caregiving and the meanings attached. In that question caregivers were asked to identify some of the difficult things about caregiving: “*For you, what are some things that are difficult about caregiving*?”

### Analysis

A mixed methods analysis was undertaken to provide additional coverage [16]. Each method of analysis has a separate purpose matching the strength of that method and plays a separate relatively self-contained role. We undertook separate parallel analysis of quantitative and qualitative data for the same cohort of patients *n* = 81. In this paper the results are presented separately, with integrated interpretation in the discussion narrative.

Descriptive statistical analyses were performed using SPSS v22 [20] to describe characteristics of the caregiver cohort, and measures of quality of life, psychological distress and subjective burden; multiple linear regression investigated predictors of caregiver burden.

Data analysis software NVivo 10 [21] was used to collate and manage the qualitative data. Inductive thematic analysis was used to identify, analyse and report themes from the caregiver responses to the open-ended question in a multi-phase process including initial coding, theme development, review and definition [22]. Initial coding of the qualitative data was carried out by two coders (MG, CM), and finalised codes and constructed themes were reviewed with a member of the research team involved in clinical management of ALS and Palliative Care (BC). As inductive thematic analysis was used, the coding was data-driven, and while there was some interpretation at the stages of theme generation and refinement, neither were driven by burden theories.

## Results

### Caregiver characteristics: descriptive statistical analysis

A majority of this caregiver cohort was female (70 %) and spousal caregivers (72 %), adult children constitute 22 %. Eighty-three percent of all caregivers lived with the person with ALS for whom they provide care. The average age of these caregivers was 55 years, ranging from 25 to 76. Thirty-two percent completed their education at degree level or higher. Caregivers spent an average of 47 h per week providing care (median 26.5 h), 44 % were working at the time of the interview, and 85 % described their own health as good to excellent (Appendix 2, Table 2).

On a scale 0 ‘*bad*’ to 10 ‘*excellent*’ the mean quality of life (MQol-SIS) for this group was 5.7. The mean anxiety score (HADS-A) was 9.6 and a mean depression score (HADS-D) of 5.9. Scores of 0–7 in either subscale are within a normal range, 8 or over is indicative of possible clinical levels of anxiety and/or depression [23]. Total psychological distress (HADS –T) was 15.4 (sd 7.1), a cut off of 12 indicates probable psychological distress [24].

#### Caregiver burden

The Zarit Burden Interview (ZBI) is a self-report instrument and assesses burden associated with patient’s behaviour and functional impairment and the impact of caregiving on caregivers’ lives in areas such as health, relationships and finances [25]. The higher the total score (scale of 0–88) the higher the level of perceived burden. Among this cohort the mean global burden score (ZBI) was 27.1 (sd 14.7), and applying a statistically derived cut-off score of ≥24 [13, 26], (Appendix 1, Table 1) indicates that 52 % of this caregiver cohort were in the ‘high burden’ category at their baseline interview.

The distribution of scores across the 22 ZBI items and mean scores for each item, are presented in Table 3 (Appendix 3). Fear of the future (*m* = 3.01), feeling that their relative is dependent on caregiver (*m* = 2.77), competing responsibilities (*m* = 1.88), impact on social life (*m* = 1.60) and time restriction (*m* = 1.55) are particular contributors to burden. Items reflecting embarrassment (*m* = 0.38), discomfort (*m* = 0.45), wishing they could leave the care to someone else (*m* = 0.53) and feeling unable to care for much longer (*m* = 0.65) have the lowest mean item scores.

Responses were skewed toward the higher frequencies for issues such as fear, dependency, and balancing responsibilities. Seventy-three percent of people said they were afraid of what the future holds for their relative, ‘quite frequently’ or ‘nearly always’; and similarly 62 % of respondents felt their relative was dependent on them as caregivers.

Multiple linear regression was calculated to predict burden. In addition to basic caregiver characteristics, the variables used were based on relevant research literature [3, 27–30]; caregiver age, sex, relationship to patient, if lives with the patient, hours of care provided per week, quality of life and psychological distress. A significant regression equation was found (R^2^ = 0.530, F (7, 64) 10.293, *p* = 0.000), with 53 % of the variance is explained by the predictor variables. Caregiver age, sex, relationship to patient and living arrangement were not found to be statistically significant predictors. Significant associations were found between caregiver burden and hours of care provided (*p* = 0.012), reduced quality of life (*p* = 0.014) and increased psychological distress (*p* = 0.004).

However, single global burden scores may mask different dimensions of burden and similar overall burden scores can translate into different caregiving experiences. Researchers have distinguished dimensions within the ZBI, and although the composition across studies varies, two factors or dimensions are consistently identified which reflect psychological distress (Personal Strain) and the impact on the caregiver’s life in general (Role Strain) and a third smaller factor Guilt or Self-Criticism has been also been found [31, 32].

Burden for each caregiver contains varying proportions of Role Strain (e.g. feeling the patient is dependent on the caregiver, social life has suffered, loss of control of own life since the person’s illness); Personal Strain (e.g. anger, discomfort, strain, and embarrassment experienced by the caregiver); and Guilt (e.g. feeling s/he should be doing more and a better job in caring for the patient).

**Fig. 1:**
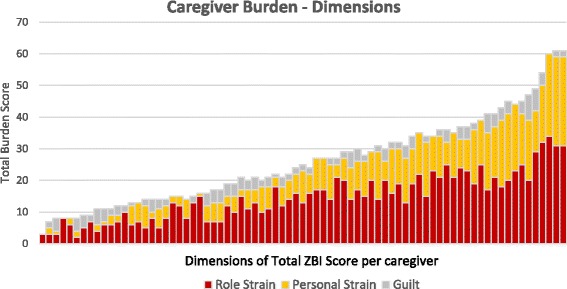
Dimensions of Caregiver Burden ZBI

The global ZBI Burden Score in ascending order, with its three dimensions Role Strain, Personal Strain and Guilt [31] for each caregiver, are shown in Fig. 1.

### Caregiver experiences: thematic analysis of qualitative data

Caregivers were asked an open-ended question about difficult aspects of caregiving. Some reported that for them there was “*nothing difficult*” about caregiving, or that the person with ALS was not in need of care, and others felt they were coping in their current situation.

**Fig. 2:**
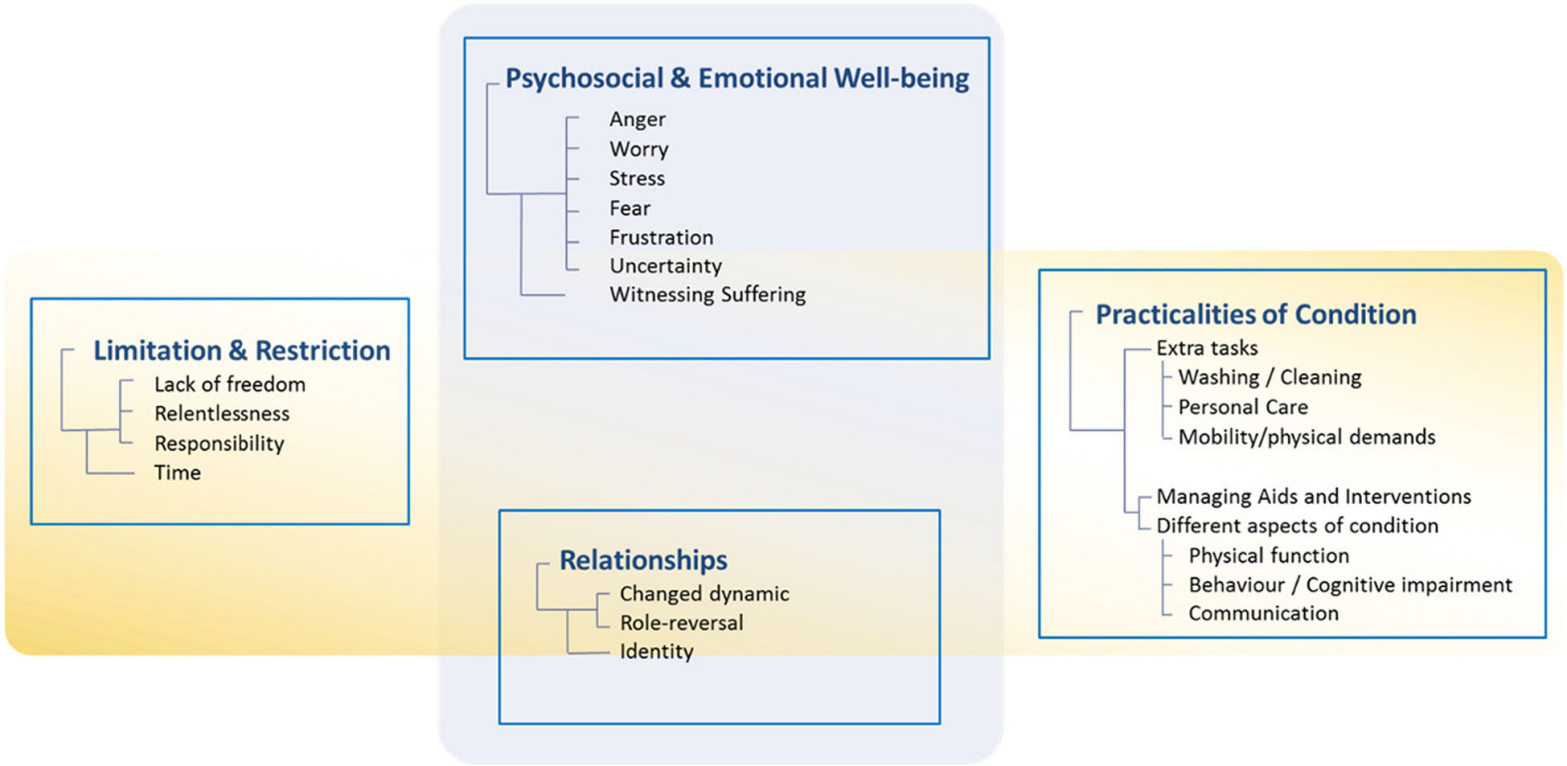
Themes and subthemes

From the difficulties identified, four main themes with related subthemes were developed. The themes centred around managing the practicalities of the ALS condition; the impact on the psychosocial and emotional Wwellbeing of the caregiver; limitation and restriction; and the effect on relationships with self and others. The four main themes and related subthemes developed through the qualitative data analysis are presented in Fig. 2.

#### Practicalities of the condition

The difficulties associated with managing a rapidly evolving disabling condition related to patients’ functional status, personality and behaviour, and communication difficulties, use of aids and appliances and the extra caregiving tasks required.

Declining physical function, reduced mobility, impaired cognition/behaviour and communication contribute to difficult experiences for the caregiver. The extra tasks included assistance with activities of daily living, washing, cleaning and personal care “*if he has to go to the toilet I think ‘Oh my god’; sometimes he doesn't make it in time; I struggle through that*” (female spouse/partner, 75 years). Due to increased physical demands on the caregiver, the physical effort of lifting, and transfers for patients with mobility difficulties. Managing aids and appliances such as wheelchairs, foot splints, and interventions such as mechanical ventilation and feeding devices can present difficulties.

#### Psychosocial and emotional wellbeing

Caregiving impacts on the psychosocial and emotional well-being of the caregiver. Participants indicated anger, worry, stress, fear, frustration, and uncertainty associated with providing care.

There can be frustration at the inability to restore previous quality of life of the person with ALS, and limited communication with the person with ALS, as illustrated by the quote: …“*I now understand housewives being frustrated about not being able to have proper adult conversations, it is always [like] caring for an infant*” (son, 46 years).

A variety of emotions are involved, “*some days I am happy and willing to take on things, and other days I am angry*” (female spouse/partner, 47 years). The emotional aspects of providing care were experienced as sometimes more difficult than the physical ones. One patient’s son felt uncomfortable with the emotional needs of his mother as she “*wants more conversations and discussions, I have a more practical approach to caregiving, she needs more of a friendship type of thing*” (Son, 48 years).

The lack of acknowledgement for efforts made could be both frustrating and hurtful, this was summed up as “*lack of understanding from* [patient] *of the burden placed on me…*” (Female spouse/partner, 66 years), and “*the pressure that my mam [patient] puts me under, her expectations* …” (Daughter, 42 years).

There was a sense of uncertainty and fear about the future “*emotional, practical and financial*” (female spouse/partner, 51 years), of not knowing what is likely to happen or how to cope with what lies ahead: “*not knowing how long the process is, and for how long he [patient] is in this state. I would like to know what’s down the road for us and what we can do*” (female spouse/partner, 70 years). For some there can be uncertainty over their own capabilities as a caregiver and others are unsure if the care provided is adequate or sufficient.

The psychological and emotional impact of exposure to suffering and deterioration of the person with ALS is a major subtheme in this analysis… “*just seeing her distress, that’s the worst part. Only having so many things to say to comfort her*” (male spouse/partner, age 65 years). There is emotional distress at witnessing the loss to the person with ALS and then also the loss of that person to the caregiver, for instance: “*it’s upsetting seeing her not being able to talk, seeing her feeling lost and isolated and not having a conversation with her*” (daughter, 41 years). The patient’s condition deteriorates and the caregiver loses the person they knew and the life they may have shared together, “*watching him go downhill, losing power in his arms and legs. It’s upsetting for you to see your husband going like that. The fact that he has his mind, that he knows what's happening to him, it’s unbelievable*” (female spouse/partner, 52 years).

#### Limitation and restriction

The relentless and usually time-delimited course of ALS is reflected in the theme of Limitation and Restriction. Providing care and being a caregiver can be restrictive in time and place. A lack of freedom means “*I have to be around, I can’t be away from the house for too long. I have my phone always!*” (Male spouse/partner 67 years).

The relentless aspect of the caregiving role was summed up as “*it just never stops. There’s always something. You never feel that there’s time to rest*” (female spouse/partner, 41 years), plans are postponed and a way of life changes “*I haven’t had any holidays and [am] afraid to go far away. It’s a constant call on your person - having to be around, having to be available*” (female, other family/relative, 70 years).

Increased demands on time, make it a limited and restricted commodity, time is taken from the caregiver and given to the patient “*the time you have to give, the time you don’t have for yourself*” (female spouse/partner, 71 years).

The responsibility of caring is also restrictive and means “*being responsible for anything and everything, having to check and think of everything. There’s so many things…*” (Female spouse/partner, 68 years). Caregiving responsibilities, especially for adult children or other relatives who provide care, often compete with their work and/or other family commitments.

#### Relationships - self and others

Providing care and assuming the role of caregiver impacts on relationships, can alter family dynamics, and how caregivers relate to other people and to themselves. Referring to her family and siblings, one daughter commented that now “*there is an element of who is minding mother [patient] best*” (daughter, 52 years). Caregiving changes existing relationships, and presents new expectations and role reversals “*that it is my mum. I shouldn’t have to be in the bathroom with her*” (daughter, 28 years). It is hard for some to reconcile the care provider and care recipient relationship with their prior relationship “*The role of a caregiver takes over, then wife. You forget you’re not his minder, you’re his wife*” (female spouse/partner, 44 years).

Identity, role change and for some, role reversal, can be accompanied by a change in how the caregiver sees him/herself: “*he is my husband, yet I am now becoming his carer. It changes how I see him or how he sees me*” (female spouse/partner, 47 years). Coming to terms with ‘becoming a carer’ and its impact on self-perception, was expressed by one respondent who explained “*you become insignificant as a person yourself, you lose your sense of identity, you’re defined as patient’s wife and MND. You nearly don’t know how to behave anymore, you’ve had to become somebody else*” (female spouse/partner, 45 years).

### Integrating findings

There are both common aspects and areas of divergence in the findings. The difficulties described by the caregivers in their open responses illustrated the physical, psychosocial and emotional difficulties associated with being a caregiver, and may be seen to reflect the Zarit Item scores and its dimensions in terms of Role and Personal Strain. Open-ended responses such as “*[I am] Not sure if I am doing the right things. Am I doing enough?*” and “*Feel guilty if not here*” point to the existence of the third dimension of Guilt/Self-criticism.

A mean global burden score (27.1) indicated that a majority of participants was in a clinically high burden category. The distribution of item scores within the ZBI (scale 0–4) point to some of the drivers of burden for this cohort of informal caregivers. These are not systematically reflected in the caregivers’ reports of difficulties experienced, however many of the burden items resonated in the open- ended responses. Fear of the future, feelings of dependency, competing responsibilities, time demands and restriction on the social life of the caregiver were drivers of burden in the ZBI and also self-identified difficulties in the open-ended responses.

Item 22 (Z22) asks “Overall how burdened do you feel in caring for your relative?” and 67 % consider themselves ‘not at all burdened’ or ‘a little burdened’. In the open-ended question some people responded that they found nothing difficult about caregiving, and while they were expecting things to get more difficult, at present things were not too bad and they were coping.

Fear of what the future holds “for your relative?” (Z7) was as a major component of burden at the item-level. Fear and uncertainty were reflected in the theme - Psychosocial and Emotional Wellbeing. The future is laced with uncertainty about coping, possible future tasks and managing the evolving condition: “*Not knowing how long the process is and for how long he is in this state. I would like to know what’s down the road for us and what we can do*”, and summed up as “*the uncertainty of the future - emotional, practical, financial*”. It was notable that the difficulties described related to the caregivers’ uncertainties and fears for their own future, aswell as the patient’s. The difficulty of witnessing suffering of the person with ALS emerged as an important issue from the open responses, but that specific area was not present as a question in the ZBI measure.

Approximately a quarter of caregivers reported that they ‘quite frequently’ or ‘nearly always’ do not have enough time for themselves (Z2) or their social life had suffered because of caring (Z12). Related concerns are found in the qualitative theme - Limitation and Restriction. This theme included difficulties regarding the relentless aspect to caregiving, time limitations, restricted freedom and constraints associated with patient dependency, for example: “…*I have to be around, I can’t be away from the house for too long*……..”; “b*eing totally tied down. She’s got to have somebody within minutes to be with her*”; “*You have to be there to keep an eye on him. It’s always on my mind. All of my time. Not that much time to myself*”.

In the ZBI caregivers were asked “Do you feel that your relative currently affects your relationship with other family members or friends in a negative way?” (Z6) however the particular complexity of relationship impacts was captured only in the open-ended responses. Here contextual pieces were provided through descriptions which highlighted the variability of relationship experiences, examples include an adult child who commented “*Now we’re back as a family with the same roles as when we were kids; there are feelings that some siblings could do more than others, there was an element of who was minding her best*”. Another man remarked that his father with ALS was not ‘the problem’ rather “*my mother, she feels the need to still be in control, she has violent outbursts at me and is angry I’m not working. There’s been a shift in the caring role and that’s changed the dynamics of the family, he [patient] is not the problem at all; I’m worried I’m going to have to look after my mother and will have no life*”. The impact of caregiving on the identity of the caregiver and how s/he sees themselves was evident in the difficulties described but not addressed in the ZBI measure.

## Discussion

Caregiver burden involves physical, psychological, emotional, social and practical challenges which can be encountered when caring for another person. A considerable amount of care for people with ALS is provided in the community by family members. These informal caregivers face both common and unique challenges, and have different caregiving experiences, which influence their ability to provide care to people with ALS.

ALS raises many of the issues relevant to caring for people with chronic disabling conditions. However several factors render the multidimensional determinants of caregiver burden uniquely complex. ALS is characterised by sudden onset, inevitable physical decline, possible cognitive and behavioural impairment, and death within 3 years. It is a relentlessly progressive condition with no cure, and currently the best treatment available to patients is optimal supportive and palliative care.

This caregiver cohort is predominantly female, and spouses/partners or adult children of the person with ALS. At their interview some people indicated that they found nothing difficult about caregiving, and when asked “overall how burdened to you feel..?” a majority indicated ‘a little’ or ‘not at all’ burdened.

Nevertheless, a mean global burden score (27.1) identified caregivers in need of further assessment and intervention, and categorically placed a majority of participants in a high burden group. Significant association was found between caregiver burden and hours of care provided, reduced quality of life and increased psychological distress. The dimensions of burden for each caregiver in terms of Role Strain, Personal Strain and Guilt and their proportionality can vary with individual caregiving circumstances. The distribution of scores across the 22 ZBI items and mean item scores indicate fear of what the future holds for the patient, feeling that their relative is dependent on them, competing responsibilities and time restriction as particular contributors to burden. Many of these burden items found expression in the open- ended responses. While some difficulties associated with emotional suffering and changed identity emerged from the open responses only. The analysis of qualitative data provided a complementary perspective and expanded on issues identified from the quantitative data and revealed new aspects.

The self-identified difficulties clustered around four main themes with related sub-themes. The caregiving role and tasks associated with management of the condition; psychosocial and emotional impact as anxiety was manifested in worry, fear and frustration, the emotional impact of witnessing suffering accompanied by an anticipatory grief at current and future losses. These caregivers are providing care, and experiencing another person’s illness, they are exposed to suffering and their own distress as a consequence. Limited time, restricted social life and extra responsibilities were all seen as difficulties associated with caregiving. The caregiving role emerges from an existing role relationship and there is a significant impact on relationships with others and also on identity. The process of ‘becoming’ and ‘being’ a caregiver can make self-identities more complex. Time is also an important locus for difficulties and emerges as a limited commodity, as time is given providing care to the person with ALS, and time is lost to the caregiver.

These findings are consistent with previous reports on caregiver burden in ALS [3, 27, 33] in terms of reduced quality of life for caregivers, increased psychological distress, fear of the future, feelings of dependency, responsibility, time demands and restriction on the social life of the caregiver. Caregivers can perform objectively similar tasks and have varied experiences [34]. People with ALS, and partners of people with ALS experience loss [35, 36]. Our findings illustrate that loss for caregivers includes empathising with the loss experienced by the patient, the loss of time to themselves, and sense of loss in the reconfiguration of the patient-caregiver relationship. Exposure to the suffering of a loved one can directly influence caregivers’ emotional experiences and subsequent psychological and physical health [12, 37].

### Mixed methods approach – an additional yield

A particular strength of this study is its mixed methods approach. The strengths of different methods are used to generate a wider variety of data than either quantitative or qualitative methods alone. This allows us to address multiple aspects of a complex construct such as burden, and adds to the conceptualization and understanding of the informal caregiver experience.

The thematic analysis reflects the main dimensions of burden in terms of psychological distress and the social consequences and impact on the caregiver’s life in general with some evidence of self-criticism. A more nuanced understanding of the impact on relationships, on roles and identity and the emotional impact of witnessing patient suffering of the patients, came through from the qualitative data. These data were analysed inductively, and as such coding and theme generation were not guided by theories of burden.

We used quantitative burden measures common in ALS caregiver research, consisting of pre-defined variables and categories. These measures produced standardised and generalizable outcomes, are useful in clinical settings as a quick way of assessing burden to identify at-risk individuals and those in need of intervention. However, they may not provide detail into the variety of elements that comprise burden at the individual level [38]. In this study the open-ended questions identified caregiving difficulties as defined by the caregivers themselves, allowed respondents to expand on their answers, provided context and a window into the intricacies of experience. This analysis points to the complexity of caregiver burden and its material consequences in peoples’ lives, and illustrates the importance of accessing differentiated information about the impact of caregiving and experiences of caregivers.

The findings have implications for policy, clinical practice and caregiver support. Effective caregiving requires that caregivers themselves receive practical and psychosocial supports. In addition to care of patients, clinicians should provide appropriate assistance to caregivers. Skills training, psychoeducation and symptom management may be of considerable benefit in reducing caregiver burden. Relationship strain, loss of intimacy, reduced social contacts and the extent of change caused by the disease may increase tension, worry and stressful situations for caregivers. It is necessary to develop methods to assess burden and caregiving difficulties to ensure adequate support and referral pathways exist. With a more comprehensive understanding of informal caregiving, health care professionals can facilitate family caregivers to articulate their emotional, psychological and care needs, and to modify initiation and delivery of interventions and services.

### Study limitations and future research

The findings relate to a group of informal caregivers of people with ALS attending a specialist multidisciplinary clinic in Dublin. Caregiving situations and experiences vary, and perceived burden and difficulties reported are for this caregiver cohort. However, while context dependent we believe these findings are relevant, and access broader processes associated with the provision of informal care.

Further analyses is required to examine predictors of burden and consider how burden and difficulties experienced vary across the key characteristics of the caregiver and the patient, over time and the course of the care-recipient’s illness.

The responses to open-ended questions as part of a semi-structured interview, have generated useful and pertinent information. However experiences of caregiving should also be explored through in-depth interviews and address the subjectivity and quality of particular experiences of caregivers. Moreover, it is important to note that caregiver burden and difficulties can be moderated and balanced by positive aspects of the caregiving situation. Work in progress includes consideration of the positive aspects of caregiving as expressed by this group of informal caregivers.

## Conclusion

Caregiver burden is rarely deconstructed in ALS. Informal caregivers in ALS face the demands of coping with a rapidly evolving care situation, physical and cognitive/behavioural decline of the care-recipient and their own physical, psychological and emotional health. The multi-layered nature of the burden phenomenon with material, physical, social, emotional and psychological components has been illustrated.

Deconstructing caregiver burden can help clinicians and health care practitioners to focus interventions on specific aspects which can vary with the caregiving situation. A comprehensive understanding of burden and the difficulties experienced is important for caregiver wellbeing at personal and existential levels, as care providers to someone with ALS, and a part of a system of care with the patient and health care professionals.

An increased understanding of the components of caregiver burden and experiences of caring allows the possibility for better supporting the caregiver, assessing the care environment and the impact on the person with ALS.

## Appendix 1

**Table 1:** Measures and Scales

Psychological Distress - Depression and Anxiety. The Hospital Anxiety and Depression Scale (HADS) [18] is composed of two 7-item subscales, with a four point ordinal response format (0–3), assessing Anxiety (HADS-A) and Depression (HADS-D). Possible scores from 0 to 21 on each subscale [23]. A score of 0–7 in either subscale indicates a normal range, 8 to 10 a possible clinical level, and 11 or over is indicative of probable clinical level [23]. The use of a summed HADS total score (HADS-T) is seen as an adequate estimate of general psychological distress [39].
Quality of Life The McGill Quality of Life Single Item Scale (MQol SIS) [19], is a single item numerical rating scale (0–10) constructed to measure self-reported quality of life (QoL). The respondent is asked to consider all parts of his/her life: physical, emotional, social, spiritual, and financial and to indicate whether over the past two days their quality of life has been - 0 ‘Very Bad’ to 10 ‘Excellent’. Higher scores are indicative of greater subjective wellbeing and quality of life.
Caregiver Burden The Zarit Burden Interview (ZBI) [17] is a widely used instrument for measuring subjectively assessed caregiver burden. The ZBI is composed of 22 items rated on a 0–4 scale, with a maximum score of 88. The global score is the sum of all item scores, higher scores indicate greater caregiver burden. ZBI cut-off scores which range from 24–26 are useful in identifying caregivers at risk of depression and in need of further assessment and intervention [26]; caregivers with a score of ≥24 are categorically defined as being in a clinically ‘high burden’ group, as this methodology has accurately predicted determinants of caregiver burden in ALS [13].
Burden dimensions: Role Strain comprises 9 ZBI items with a possible range of 0–36; Personal Strain comprises 10 ZBI items with a possible range of 0–40; Guilt comprises 2 ZBI items with a possible range of 0–8. The ZBI item 22 which asks “Overall, how burdened do you feel in caring for this person?” was not included in this analysis. This item provides a global estimate of burden and loaded high on both factors Personal and Role Strain [31].

## Appendix 2

**Table 2:** Caregiver Characteristics and Outcomes

Caregivers (*N* = 81)				
Respondent Age				
Mean	54.9 (sd 13.4)			
Range	25–76 Years			
Sex				
Male	24		29.6 %	
Female	57		70.4 %	
Do you live with the Patient				
Live with Patient	67		82.7 %	
Do not live with Patient	14		17.3 %	
Relationship to the patient				
Spouse/partner	58		71.6 %	
Son/daughter	18		22.2 %	
Parent	2		2.5 %	
Sibling	2		2.5 %	
Friend	1		1.2 %	
Highest level of education completed				
Primary education	6		7.4 %	
Secondary	30		37.0 %	
Technical	13		16.0 %	
Degree or higher	26		32.1 %	
Other	6		7.4 %	
Principal economic status				
Working for payment or profit	36		44.4 %	
Unemployed	4		4.9 %	
Looking after family/home	18		22.2 %	
Retired	21		25.9 %	
Unable to work due to permanent sickness or disability	2		2.5 %	
Hours of care provided per week				
Mean	46.9 (sd 48.5)			
Median	26.5			
Range	0–168 h			
In general, would you say your health is:				
Excellent	16		19.8 %	
Very good	28		34.6 %	
Good	25		30.9 %	
Fair	10		12.3 %	
Poor	2		2.5 %	
Caregiver Burden				
High Burden	40		51.9 %	
Low Burden	37		48.1 %	
Measures and Scales	Mean	St Dev	Median	Range
HADS-T Total Distress	15.4	7.1		
HADS-Anxiety	9.6	4.3		
HADS-Depression	5.9	3.8		
MQoL SIS Quality of Life	5.7	2.1		
ZARIT Burden Total	27.1	14.7	26.0	3–65
ZBI Role Strain^a^	14.8	7.6	14.5	2–34
ZBI Personal Strain^a^	8.7	6.8	7.0	0–28
ZBI Guilt^a^	2.4	1.8	2.0	0–8

^a^[31]

## Appendix 3

**Table 3:** Zarit Burden Interview Items – distribution of scores

			Score					
			0	1	2	3	4	
ZBI Item	Question	*N*	Never	Rarely	Sometimes	Quite frequently	Nearly always	Mean Item Score
1	Do you feel that your relative asks for more help than he or she needs?	78	30	20	19	5	4	1.14
2	Do you feel that because of the time you spend with your relative, you don’t have enough time for yourself?	78	25	10	24	13	6	1.55
3	Do you feel stressed between caring for your relative and trying to meet other responsibilities for your family or work?	76	16	8	27	19	6	1.88
4	Do you feel embarrassed about your relative’s behaviour?	78	59	11	6	1	1	0.38
5	Do you feel angry towards your relative when you are around him/her?	78	39	17	20	1	1	0.82
6	Do you feel that your relative currently affects your relationship with other family members or friends in a negative way?	78	39	11	20	5	3	1.00
7	Are you afraid of what the future holds for your relative?	78	2	3	16	28	29	3.01
8	Do you feel your relative is dependent upon you?	78	5	7	18	19	29	2.77
9	Do you feel strained when you are around your relative?	78	30	14	28	5	1	1.14
10	Do you feel your health has suffered because of your involvement with your relative?	78	41	16	15	5	1	0.83
11	Do you feel that you don’t have as much privacy as you would like because of your relative?	78	46	7	16	5	4	0.90
12	Do you feel that your social life has suffered because you are caring for your relative?	78	24	11	23	12	8	1.60
13	Do you feel uncomfortable about having friends over because of your relative?	78	61	7	4	4	2	0.45
14	Do you feel that your relative expects you to take care of him/her as if you were the only one he/she could depend on?	77	28	13	15	6	15	1.57
15	Do you feel that you don’t have enough money to care for your relative in addition to the rest of your expenses?	78	43	10	12	6	7	1.03
16	Do you feel that you will be unable to take care of your relative for much longer?	78	47	14	14	3	0	0.65
17	Do you feel you have lost control of your life since your relative’s illness?	78	30	11	21	11	5	1.36
18	Do you wish you could just leave the care of your relative to someone else?	77	53	10	12	1	1	0.53
19	Do you feel uncertain about what to do about your relative?	77	28	21	22	5	1	1.09
20	Do you feel you should be doing more for your relative?	78	28	18	27	3	2	1.14
21	Do you feel you could do a better job in caring for your relative?	78	22	20	32	3	1	1.24
		*N*	Not at all	A little	Moderately	Quite a bit	Extremely	Mean Item Score
22	Overall how burdened do you feel in caring for your relative?	78	27	25	11	12	3	1.22

## Additional file

Additional file 1: Extracts from semi-structured interview guide and standardised questionnaires. (DOCX 16 kb)

## Abbreviations

ALS: amyotrophic lateral sclerosis
HADS: hospital anxiety and depression scale
HADS- A: hospital anxiety and depression scale - anxiety
HADS- D: hospital anxiety and depression scale - depression
HADS- T: hospital anxiety and depression scale - total psychological distress
MND: motor neuron disease
MQoL SIS: McGill Quality of Life Single Item Scale
ZBI: Zarit Burden Interview
