# Supplementary material for: Caregiving in ALS – a mixed methods approach to the study of Burden
Source: BMC Palliat Care. 2016 Sep 5;15(1):81. doi: 10.1186/s12904-016-0153-0 (PMC5011853; doi:10.1186/s12904-016-0153-0)
Supplement: Additional file 1: — Extracts from semi-structured interview guide and standardised questionnaires. (DOCX 16 kb) [file 12904_2016_153_MOESM1_ESM.docx]

| **Additional file 1**  **Extracts from semi-structured interview guide and standardised questionnaires** | |
| --- | --- |
|  |  |
| **A population based analysis of palliative needs, services and outcomes in Motor Neuron Disease** | |
| **Caregiver interview** | |
|  |  |
| Date of Interview |  |
| Location of Interview |  |
| Who is present at the time of the interview? |  |
| Start time for Interview |  |
|  |  |
| **Part 1** | |
| **Respondent Demographic details** | |
| Sex | Male/Female |
| Date of Birth (dd/mm/yyyy) |  |
| Marital Status | Single |
|  | Married |
|  | Living with partner |
|  | Separated |
|  | Divorced |
|  | Widowed |
| What is the highest level of education that you have completed? | No formal education |
|  | Primary education |
|  | Secondary education |
|  | Technical or vocational |
|  | Degree |
|  | Postgraduate |
|  | Other |
| What is your relationship to (patient name)? | Spouse/partner |
|  | Son/daughter |
|  | Parent |
|  | Sibling |
|  | Friend |
|  | Other |
| Do you live with (patient name)? | Yes/No |

|  |  |
| --- | --- |
| **Respondent Socio-economic details** | |
| How would you describe your present principal status? | Working for payment or profit |
|  | Looking for first regular job |
|  | Unemployed |
|  | Student/pupil |
|  | Looking after home/family |
|  | Retired from employment |
|  | Unable to work due to permanent sickness or disability |
|  | Other |
| In general, would you say your health is….? | Excellent |
|  | Very good |
|  | Good |
|  | Fair |
|  | Poor |
| How many hours of care do you provide to (patient name) per week? |  |
|  |  |
| **Part 2: Standardised Questionnaires** | |
| **McGill Quality of Life questionnaire** |  |
| Cohen SR, Mount BM, Strobel MG, Bui F. The McGill Quality-of-Life Questionnaire - a measure of quality-of-life appropriate for people with advanced disease - a preliminary-study of validity and acceptability. Palliative Medicine. 1995;9(3):207-19. doi:10.1177/026921639500900306. | |
|  |  |
| Contact information: Dr. Robin Cohen, Research Director, Palliative Care, Jewish General Hospital, Pavilion H, 3755 Côte Ste Catherine, Montreal,QC, Canada H3T 1E2 | |
| **Hospital anxiety and depression scales (HADS)** | |
| Zigmond AS, Snaith RP. THE HOSPITAL ANXIETY AND DEPRESSION SCALE. Acta Psychiatrica Scandinavica. 1983;67(6):361-70. doi:10.1111/j.1600-0447.1983.tb09716.x. | |
| **Zarit Burden Interview** |  |
| Zarit SH, Reever KE, Bachpeterson J. RELATIVES OF THE IMPAIRED ELDERLY - CORRELATES OF FEELINGS OF BURDEN. Gerontologist. 1980;20(6):649-55. | |
|  |  |
| Contact information and permission to use: MAPI Research Trust, Lyon, France. E-mail: PROinformation@mapi-trust.org – Internet: www.mapi-trust.org | |
| **Caregiver open-ended question:** |  |
| *For you, what are some things that are difficult about caregiving?* | |
|  |  |
| End time for Interview |  |
